# Opportunities, Challenges and Pitfalls of Using Cannabidiol as an Adjuvant Drug in COVID-19 †

**DOI:** 10.3390/ijms22041986

**Published:** 2021-02-17

**Authors:** Barbara Malinowska, Marta Baranowska-Kuczko, Aleksandra Kicman, Eberhard Schlicker

**Affiliations:** 1 Department of Experimental Physiology and Pathophysiology, Medical University of Białystok, 15-222 Białystok, Poland; mabar@umb.edu.pl (M.B.-K.); olakicman@gmail.com (A.K.); 2 Department of Clinical Pharmacy, Medical University of Białystok, 15-222 Białystok, Poland; 3 Department of Pharmacology and Toxicology, University of Bonn, 53127 Bonn, Germany

**Keywords:** ACE2, cannabidiol, COVID-19, SARS-CoV-2, respiratory disease

## Abstract

Severe acute respiratory syndrome coronavirus 2 (SARS-CoV-2) infection may lead to coronavirus disease 2019 (COVID-19) which, in turn, may be associated with multiple organ dysfunction. In this review, we present advantages and disadvantages of cannabidiol (CBD), a non-intoxicating phytocannabinoid from the cannabis plant, as a potential agent for the treatment of COVID-19. CBD has been shown to downregulate proteins responsible for viral entry and to inhibit SARS-CoV-2 replication. Preclinical studies have demonstrated its effectiveness against diseases of the respiratory system as well as its cardioprotective, nephroprotective, hepatoprotective, neuroprotective and anti-convulsant properties, that is, effects that may be beneficial for COVID-19. Only the latter two properties have been demonstrated in clinical studies, which also revealed anxiolytic and antinociceptive effects of CBD (given alone or together with Δ^9^-tetrahydrocannabinol), which may be important for an adjuvant treatment to improve the quality of life in patients with COVID-19 and to limit post-traumatic stress symptoms. However, one should be aware of side effects of CBD (which are rarely serious), drug interactions (also extending to drugs acting against COVID-19) and the proper route of its administration (vaping may be dangerous). Clearly, further clinical studies are necessary to prove the suitability of CBD for the treatment of COVID-19.

## 1. Introduction

Coronavirus disease 2019 (COVID-19) is caused by a new virus entity, the severe acute respiratory syndrome coronavirus 2 (SARS-CoV-2) and has grown to be one of the most dangerous pandemics in the history of mankind [1,2,3]. Its death toll of more than 2.2 million (1 February 2021) people has a similar magnitude like the Asian flu (1957–1958; 1–4 million) and Hong Kong flu (1968–1970; 1–4 million) and is already higher than that of the swine flu (2009–2010; up to 0.6 million; [4]). It is responsible for significantly more fatalities than the severe acute respiratory syndrome (SARS, 2002–2004; 774 deaths) and Middle East respiratory syndrome (MERS, 2012–present; 935 deaths) pandemics caused by the SARS-CoV and MERS-CoV viruses, respectively [4]; the latter two, like SARS-CoV-2, belong to the *Coronaviridae* family [5].

Vaccines protecting against SARS-CoV-2 have become available within a year of its emergence and they appear to be effective and safe [6,7]. Although general vaccination began in December 2020 in many countries, it will take months until the number of vaccinated people is high enough to provide herd immunity [6]. Therapeutic approaches are largely symptomatic and supportive. Many drugs have been examined in clinical studies, mostly with disappointing results [2,3,8,9]. Nonetheless, the antiviral drug remdesivir shortens hospitalization time and the glucocorticoid dexamethasone even reduces mortality [10,11]. In addition, it is suggested that alternative compounds, including phytochemicals and natural agents targeting coronavirus development directly or as a result of their immunomodulatory effects, could be applied as potential therapies and for the prevention of COVID-19 [12,13,14,15].

The two senior authors of the present article (B.M. and E.S.) serve as editors of a Special Issue of *Int. J. Mol. Sci.* dedicated to research on cannabidiol (CBD), which has been suggested as a putative drug against COVID-19 but the views of different investigators on this compound have been found to vary greatly. Thus, we decided to conduct a review on the advantages and disadvantages of using CBD as a potential agent for the prevention and treatment of COVID-19 based on scientific reports on its influence on selected disease models and in clinical studies. We also summarize current knowledge about the effects of this compound on SARS-CoV-2 infection. The aim of the present review is to examine the suitability of CBD (Section 3) as an antiviral drug against SARS-CoV-2 (Section 4) and as an agent for the prevention and treatment of disease states in the preclinical (Section 5) and clinical (Section 6) settings. The review ends with a synopsis of the pros and cons of using CBD as a potential drug to treat COVID-19 (Section 7).

## 2. Mode of Infection and Symptoms of COVID-19

**Figure 1:**
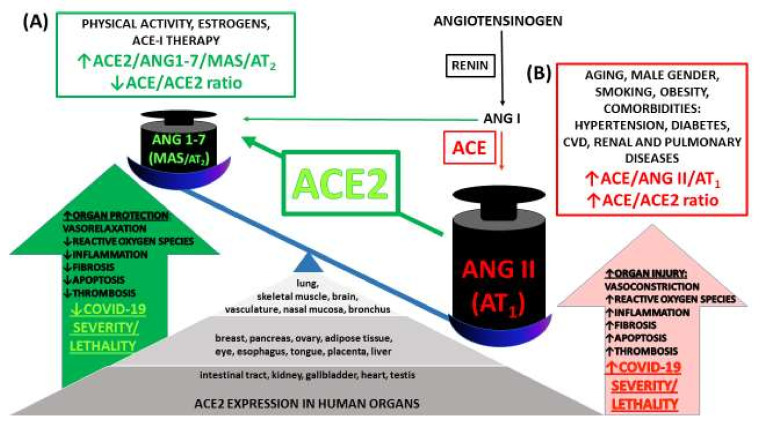
Expression of angiotensin-converting enzyme 2 (ACE2) in human tissues and organs, its counter-regulatory effects on the ACE → Ang II → AT_1_ axis and interaction with coronavirus disease 2019 (COVID-19). ACE2 is ubiquitous and widely expressed in many organs targeted and damaged by COVID-19 caused by severe acute respiratory syndrome coronavirus 2 (SARS-CoV-2). It is a membrane-bound enzyme and an endogenous counter-regulator of the renin-angiotensin hormonal cascade. It degrades angiotensin II (Ang II) to angiotensin 1-7 (Ang 1-7) that exerts beneficial effects opposed to those of Ang II. Ang 1-7 acts through the G protein-coupled receptor MAS and, to a lesser extent, Ang II type 2 receptors (AT_2_). ACE and ACE2 and their major products, Ang II and Ang 1-7, respectively, are linked in almost a ying/yang process, that is, when one decreases, the other increases and vice versa [18]. Thus, reduced activity of the deleterious ACE → Ang II → Ang II receptor type 1 (AT_1_) axis (red) is coupled with increased activity of the protective ACE2 → Ang 1-7 → MAS receptor axis (green). A lower ACE/ACE2 ratio (**A**) (occurring in women, in exercise-trained individuals and patients well-treated with ACE inhibitors (ACE-I)) leads to beneficial effects such as vasorelaxation, anti-inflammatory, anti-oxidative, anti-fibrotic and anti-thrombotic effects that predispose towards a lower risk of cardiovascular disease (CVD) and better COVID-19 outcomes. By contrast, a high ACE/ACE2 ratio (**B**) that is increased in males, elderly and many pathologies (especially CVD, pulmonary and renal diseases and obesity) may aggravate COVID-19 infection [19,20,21,22].

Infection with SARS-CoV-2 occurs mainly by aerosol/droplet transmission through direct contact with an infected person. The virus enters the body through the epithelial cells of the tongue, bronchi and lungs after attaching to angiotensin-converting enzyme 2 (ACE2). An important function of membrane-bound and soluble ACE2 is degradation of angiotensin II (Ang II) to angiotensin 1-7 (Ang 1-7), which exerts a beneficial influence, as opposed to numerous detrimental effects of high concentrations of Ang II (for details, see Figure 1 and Supplementary Table S1). The affinity of SARS-CoV-2 to ACE2 is determined by glycoprotein S1 localized on the characteristic viral “spikes.” S1 binds to the enzyme via the receptor-binding domain. Transmembrane serine protease 2 (TMPRSS2), which allows the entry of the virus into the cytoplasm of host cells [1,2,5,16], also plays a significant role in COVID-19 infection. Moreover, quite recently, it was shown that the membrane protein neuropilin-1 (NRP1) promotes SARS-CoV-2 entry [17].

COVID-19 is usually asymptomatic. In most symptomatic patients, SARS-CoV-2 infection is mild with symptoms including fever, shortness of breath, coughing, fatigue, anosmia, ageusia and muscle pain. Less common symptoms are nausea, vomiting and diarrhea [1,2,16,23]. The risk of developing a severe form of the disease increases with male gender, age and smoking; a further aggravation occurs in people with comorbidities such as hypertension, diabetes, obesity, cardiovascular or chronic respiratory system diseases, in which the concentration of the soluble form of ACE2 is substantially higher (Figure 1). By contrast, in healthy individuals ACE2 activity is much lower or not detected [24,25].

**Figure 2:**
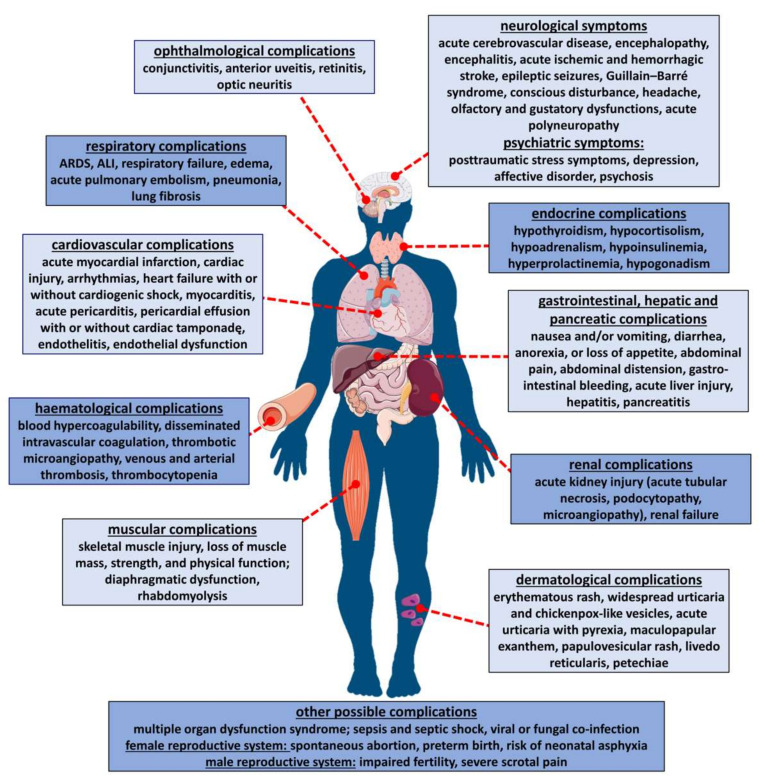
Systemic manifestation of COVID-19 infection. For respective literature, see Section 2. ALI, acute lung injury; ARDS, acute respiratory distress syndrome.

Because ACE2 is ubiquitous and widely expressed in the heart, blood vessels, gut, lungs (particularly in type 2 pneumocytes and macrophages) and in different types of cells (for details, see Figure 1 and Supplementary Table S1), COVID-19 is a multiple-organ disease, as summarized in Figure 2. One of the most common complications is an acute respiratory distress syndrome (ARDS) resulting from the cytokine storm phenomenon, a violent and uncontrolled inflammatory reaction in response to the presence of the virus in the host organism [1,2,16,23]. However, the negative consequences of COVID-19 are also connected with other disorders of the respiratory system [1,16,26,27], with the cardiovascular system (for review, see [1,16,22,26,28,29,30]) and with dangerous hematological complications, particularly thromboembolism [1,16,31,32,33,34,35,36]. In addition, pathological changes in the renal [1,16,27,33,37,38], gastrointestinal, hepatic, pancreatic [1,16,27,31,39,40] and nervous systems [1,27,41,42,43] have been described. Disturbances of the eyes [1,44], endocrine [45,46] and reproductive functions [31,45,46,47], skeletal muscles [1,33,48] and skin [1,49,50] occur as well (for details, see Figure 2).

## 3. Cannabidiol—Pharmacological Potential and Mechanism of Action

Non-intoxicating CBD is a phytocannabinoid isolated from the *Cannabis sativa* plant [13,14] and, apart from the psychoactive ∆^9^-tetrahydrocannabinol (THC), represents the best-studied compound in this group.

Numerous studies have demonstrated a range of beneficial properties associated with CBD, including anti-inflammatory, antioxidant, antiarthritic, cardio- and neuroprotective, anticonvulsant, procognitive and analgesic effects (for detail, see reviews, for example, [51,52,53]). At the moment, there are two indications. CBD (Epidiolex^®^) has been approved for the treatment of intractable childhood-onset epilepsy (Dravet and Lennox-Gastaut syndrome). Its combination with THC (nabiximols (Sativex^®^); 100 µL of oromucosal spray contains 2.5 mg CBD and 2.7 mg THC) has been approved for the therapy of spasticity in multiple sclerosis. Moreover, a potential therapeutic effect of CBD is suggested for inflammatory and autoimmune diseases, anxiety disorders, schizophrenia, depression, Alzheimer’s disease, Parkinson’s disease, chronic pain, cancer and diabetic complications [54,55].

With respect to the potential use of CBD in COVID-19, sixteen publications have so far appeared. Four publications are experimental (see Section 4) whereas another twelve discuss data from the literature.

Within the group of papers dealing with the literature, seven publications consider the potential use of CBD for the treatment of COVID-19 mainly due to its potent anti-inflammatory activity [14,15,56,57,58,59,60]. However, as suggested by another three authors, there is a lack of high-quality studies dedicated to the anti-inflammatory effects of CBD [61,62] or to its effects on the central nervous system (e.g., anxiety or neurological complications) [63]. Two authors warn that the anti-inflammatory action of CBD might exert a potential detrimental effect on the immune system even leading to enhancement of viral infections [64,65]. One should also consider that vaping CBD was associated with severe COVID-19 problems [57] and not overlook the unclear impact of “over-the-counter” CBD on the immunity of the SARS-CoV-2 infection [62]. Due to the lack of rigorous legal regulations, CBD is indeed commonly used as over-the-counter product, often of unapproved and unknown composition [51,54,55].

The multi-directional properties of CBD mentioned above arise from its complex mechanism of action. CBD has a low affinity for cannabinoid receptors (CB-Rs); it acts as a negative allosteric modulator of cannabinoid receptor type 1 (CB_1_-R) and as an inverse agonist of cannabinoid receptor type 2 (CB_2_-R). In addition, CBD acts via many other molecular targets including G-protein-coupled receptors (GPCRs; for example, activation of the peroxisome proliferator-activated γ (PPARγ) receptor and serotonin 5-HT_1A_ and 5-HT_2A_ receptors) and ionotropic receptors (e.g., activation of vanilloid TRPV1 but inhibition of serotonin 5-HT_3_ receptors). Moreover, it inhibits various transporters (e.g., adenosine uptake) and enzyme activities (e.g., fatty acid amide hydrolase (FAAH), an enzyme responsible for the degradation of the endocannabinoid anandamide) (for details and other molecular targets, see reviews [51,66]). In order to explain its effect against oxidative/nitrative stress, direct effects on the mitochondria and nuclei have been taken into consideration as additional molecular mechanisms [66].

## 4. Potential Antiviral Activity of CBD

Various effects and molecular mechanisms of CBD have been described in the previous section and some of them may be helpful in the context of the COVID-19 infection. Before discussing them in preclinical studies (Section 5) and in the clinical setting (Section 6), we would like to draw the attention of the reader to four experimental papers on the basis of which the potential usefulness of CBD against the SARS-CoV-2 virus was suggested.

In the first paper, Wang et al. [67] examined whether the gateways for the entry of the virus into cells, ACE2 and TMPRSS2, are affected by CBD. They found that high-CBD Cannabis sativa extracts decreased ACE2 and TMPRSS2 protein levels in artificial human 3D models of oral, airway or intestinal tissues primed by tumor necrosis factor α (TNF-α) plus interferon γ (IFN-γ). Extracts had different cannabinoid and terpene profiles and not all extracts under study were equally effective. In particular, pure CBD failed to affect ACE2 and TMPRSS2 protein levels in the airways, suggesting an entourage effect of the components of the extracts; the possibility that a component in the extracts other than CBD is the active principal component can so far not be excluded. Some extracts produced undesired molecular effects, that is, upregulated the levels of the ACE2 gene and protein. The idea of using high-CBD products (administered, for example, via mouth wash) to limit the entry of SARS-CoV-2 into susceptible hosts seems to be attractive but requires unambiguous scientific confirmation [56].

In the second paper (Raj et al. [68]), a direct antiviral effect of CBD was identified. The authors first screened several cannabinoids in silico and then examined CBD and THC, which appeared to have particularly promising effects in cultured Vero cells infected with SARS-CoV-2. CBD exhibited an IC_50_ value of 8 µM for its inhibitory effect on SARS-CoV-2 replication and was at least as potent, in this respect, as the antiviral drugs remdesivir, chloroquine and lopinavir, which are already used for the treatment of COVID-19 [2,3,8,9]. Again, these interesting data await unambiguous confirmation. By the way, CBD also inhibits the replication of hepatitis C virus in vitro [69] but was not active against the hepatitis B virus [69] or the Kaposi sarcoma-associated herpesvirus [70].

In the third and fourth experimental study suggesting the potential usefulness of CBD for treatment of COVID-19 [71,72] an acute respiratory distress syndrome was induced in mice by poly(I:C), a synthetic analogue of viral double-stranded RNA. Table 1 shows that CBD indeed had a beneficial effect in this condition. Chronic CBD application was also effective against the negative consequences of the infection in mice suffering from Theiler’s murine encephalomyelitis virus, which induces demyelinating disease [73,74]. However, in the aforementioned in vivo experiments, the beneficial effects of chronic CBD administration were due to its anti-inflammatory properties and not direct antiviral activities (Table 1).

## 5. Preclinical Studies on the Use of CBD for COVID-19 Treatment

As discussed in the previous section, CBD has a direct as well as an indirect antiviral effect by reducing the number of ACE2 molecules via which the SARS-CoV-2 virus enters cells. Table 1 shows that, in preclinical models, CBD has a beneficial effect in many disease states also occurring in COVID-19. We mainly concentrated on the in vivo effects of chronic CBD administration. Acute in vivo or in vitro effects were considered if they were of interest with respect to COVID-19. Table 1 differentiates between prophylactic (preventive) and therapeutic CBD administration, that is, whether CBD was given before (or simultaneously with) the stimulus leading to the disease or after the pathological state had fully developed, respectively.

First of all, we would like to underline the effectiveness of CBD found in experimental models of respiratory failure. As already mentioned in the previous section, chronic CBD administration reduced the acute respiratory distress syndrome and the cytokine storm induced by polyriboinosinic:polyribocytidylic acid (poly(I:C)), a synthetic analogue of viral RNA [71,72]. Interestingly, it increased the expression of blood apelin [72], which serves as a catalytic substrate for ACE2 [24]. Moreover, the administration (mainly intraperitoneally (i.p.)) of CBD improved lung function and reduced inflammation in experimental acute lung injury (ALI) [75,76], pulmonary hypertension [77], lung injury induced by brain hypoxic/ischemic damage [78] and asthma [79,80] (Table 1). The beneficial influence of CBD resulted mainly from its significant anti-inflammatory properties [75,76,78,79,80]. Importantly, CBD has been shown to improve lung function [76], gas exchange [77], blood oxygen saturation [77] and to reduce allergen-induced airway obstruction [81]. Undoubtedly, a favorable effect of CBD is also the strong relaxation of the human pulmonary artery determined under in vitro conditions [82]. The unequivocally beneficial action profile of CBD is, however, contrasted by the results obtained by Karmaus et al. [83], who described a proinflammatory effect of prophylactically administered CBD (once daily for 3 days) in lipopolysaccharide-induced lung inflammation in mice. Moreover, CBD does not have antitussive properties and does not affect trachea contraction [84]. In most studies, CBD (i.p. or per os (p.o.) in doses of 5–10 mg/kg per day) was administered prophylactically or therapeutically for 2–4 days only (Table 1). Only in the rat model of monocrotaline-induced pulmonary hypertension CBD was given prophylactically for 3 weeks [77].

A cardioprotective influence of CBD has been mainly shown in experiments in which prophylactic administration (predominantly in one dose given before occlusion or reperfusion) prevented the negative consequences of experimental myocardial infarction by decreasing the infarct size or arrhythmia ([85,86,87,88]; Table 1). Beneficial therapeutic effects (including improvement of cardiac systolic and diastolic dysfunction, reduction in coronary vasoconstriction, enhancement of mesenteric artery vasorelaxation and improvement of metabolic parameters) of chronic CBD administration (1 to 11 weeks) have been demonstrated in autoimmune myocarditis [89], diabetic cardiomyopathy [90], primary and secondary hypertension [91,92] and in Zucker diabetic fatty rats [93]. As in the case of respiratory failure, the favorable influence of CBD on the cardiovascular system is connected mainly with its anti-inflammatory properties and, in addition, with its antioxidative, antinitrative and antifibrotic effects (for details, see Table 1).

There is a significant association between severe COVID-19 and the occurrence of thromboembolism (for review, see [1,16,32,33,34,35,36]). Unfortunately, only a few publications have addressed the influence of CBD on the components or parameters of hemostasis. Thus, the chronic administration of CBD normalized the plasma tissue plasminogen activator and plasminogen activator inhibitor-1 enhanced by monocrotaline-induced pulmonary hypertension in rats [77]. A decrease in platelet aggregation occurred after a single CBD dose given before (but not after) myocardial infarction [87]. CBD (injected prophylactically, acutely or chronically) failed to reduce vessel thrombogenesis and did not modify human platelet aggregation when given in vitro [94].

Nephroprotective properties of CBD have been described only for its acute administration (one or maximally two doses) in experimental models of ischemic acute kidney injury [95,96,97]. They were connected with its anti-inflammatory, antioxidative and antinitrative properties (Table 1).

With respect to disorders of the gastrointestinal tract, cases of acute and chronic prophylactic CBD administration have been associated with anti-nausea and antiemetic effects in experimental nausea and vomiting induced by lithium chloride via the activation of serotonin 5-HT_1A_ receptors [98]. Since CBD antagonizes 5-HT_3_ receptors under in vivo conditions [99], its antiemetic effect might also result from the antagonism of 5-HT_3_ receptors. Hepatoprotective properties of CBD have been described in models of liver injury induced by hepatic artery and portal vein occlusion [100], chronic ethanol administration [101], thioacetamide [102] or cocaine [103]. Again, the beneficial influence of CBD is connected mainly with its anti-inflammatory and antioxidative properties. Unfortunately, with the exception of chronic ethanol administration [101], CBD was only given once. Prophylactic administration of CBD had a beneficial influence in experimental acute pancreatitis, based on its anti-inflammatory properties [104]. Moreover, its therapeutic administration at one [105] or three [106] doses was shown to reduce intestinal hypermotility (Table 1). Interestingly, cannabis extract with a high CBD content reduced inflammatory changes in the colon more strongly than CBD did alone [106].

The most common symptoms of COVID-19 include anosmia, ageusia and fever. A PubMed-based search did not identify any publications showing that CBD is useful in the case of loss of smell or taste. With respect to the nervous system, one might mention its prohedonic activity, which occurred upon chronic administration in rats that were exposed to chronic unpredictable mild stress [107]. Moreover, CBD is approved for the treatment of Dravet syndrome, a condition that features recurrent seizures triggered by fever [108]. Its effective anticonvulsant activity has been confirmed both in human (for review, see [108]) and different experimental models (for example [109,110], see Table 1). In addition, a neuroprotective influence of CBD (administered once or twice) has been determined in hepatic encephalopathy [102], perinatal hypoxia/ischemia encephalopathy [111,112], sepsis-related encephalitis (including increase in integrity of blood-brain barrier; [113]) or cerebral ischemia [114,115]. CBD (given for up to 10 days) had also a beneficial effect on encephalomyelitis and multiple sclerosis induced by Theiler’s murine encephalomyelitis virus [73,74].

Tears and the eyes in general, create portals for coronavirus entry. Although we did not find any publications regarding the potential application of CBD for conjunctivitis (the most common ocular manifestation of COVID-19), one should remember that CBD has been suggested as a putative novel therapy for diabetic retinopathy [116] and retinal inflammation ([117]; Table 1). Both protective effects are associated with the anti-inflammatory and antioxidative actions of CBD which are also beneficial in the reduction of the negative consequences of perinatal hypoxia/ischemia [111,112]. In this context, one may consider the risk of neonatal asphyxia in children from mothers suffering from COVID-19 (Figure 2).

Unfortunately, there are limited publications suggesting potential beneficial effects of CBD in endocrine, muscular and dermatological disorders, that also are listed among those related to COVID-19 (Table 1). Thus, chronic CBD administration has been shown to reduce hyperglycemia and to improve metabolic dysfunction [118,119] in experimental models of obesity/diabetes, which is important if one considers that high glucose plasma levels and diabetes are risk factors for COVID-19 [120]. Moreover, CBD was found to prevent losses in functionality due to skeletal muscle degeneration [121]. Beneficial anti-inflammatory properties of chronic and acute administration of CBD on skin function have been shown in nude rats [122] and in vitro in human sebocytes, keratinocytes and skin organ culture [123,124].

Importantly, the effectiveness of CBD has been shown in multiple organ dysfunction. Acute and chronic administration in an experimental model of sepsis reduced mortality, lipid peroxidation and oxidative damage of proteins in many vital organs [125]. However, one should keep in mind that CBD, given acutely, had a pro-oxidative effect and increased oxidative damage of proteins in the lungs ([125]; Table 1).

In summary, preclinical studies show that acute and chronic administration of CBD through prophylactic and/or therapeutical interventions has numerous beneficial effects in organs that are also targeted by the coronavirus; they mainly result from CBD’s anti-inflammatory and antioxidative actions. The precise mechanism(s) is/are still unknown although in a few studies, the involvement of cannabinoid CB_1_, CB_2_, GPR55, vanilloid TRPV1, adenosine A_2A_ and serotonin 5-HT_1A_ receptors has been described (Table 1).

## 6. Use of CBD for COVID-19 Treatment? Clinical Studies

In the ClinicalTrials.gov database (accessed on 1 February 2021), we found six clinical trials (two active, not recruiting; two not yet recruiting; and two recruiting) in which the use of CBD is being addressed in the context of COVID-19. Two studies are dedicated to the use of CBD in patients with mild to moderate symptoms, including the study entitled “Cannabidiol for COVID-19 patients with mild to moderate symptoms” (daily doses of 300 mg for 14 days) and the study entitled “Synthetic CBD as a therapy for COVID-19” (dose and duration of administration not specified). Another two studies are dedicated to patients with higher risk, including the study entitled “*Cannabidiol* treatment for severe and critical coronavirus (*COVID-19*) pulmonary infection” (daily doses of 300 mg for 14–28 days or until discharge) and the study entitled “Cannabidiol in patients with COVID-19 and cardiovascular disease or risk factors” (daily doses of 525 mg/70 kg for 28 days). The fifth study, entitled “Outcomes mandate national integration with Cannabis as medicine for prevention and treatment of COVID-19 (OMNI-Can)” will examine the efficacy and safety of using medical cannabis for chronic medical conditions, including COVID-19 (dose and duration of administration not specified). As suggested by the title “Burnout and distress prevention with cannabidiol in front-line health care workers dealing with COVID-19,” the final study is dedicated to the hospital staff rather than to patients (daily doses of 175 mg/70 kg for 28 days).

Since it will take some time until the latter studies have been completed, the question arises as to whether there are other clinical studies based on CBD that may point to its suitability for the prevention/treatment of COVID-19 (Table 2). In the ClinicalTrials.gov database (accessed on 1 February 2021), there are 186 items regarding formulations containing CBD (59 studies were signed as completed; the results of 13 of the latter trials are presented in the database and 9 of them have been published). In contrast to the results of preclinical studies (Table 1), so far, no clinical studies have demonstrated promising effects of CBD on patients with respiratory failure (Table 2). There are two publications based on a few patients with chronic obstructive pulmonary disease (COPD). CBD given acutely together with THC in vaporized form [126] or as a sublingual spray [127] had no or only minimal beneficial effects on airway function, exertional breathlessness at rest and during exercise and simulated breathlessness (Table 2). Clinical trials indicating a potential usefulness of CBD in cardiovascular, hematological and renal symptoms associated with COVID-19 could not be found.

With respect to the gastrointestinal complications related to COVID-19 (see Figure 2), the effect found for Sativex^®^ (combination of 2.7 mg THC and 2.5 mg CBD), given on top of the standard antiemetic therapy against delayed chemotherapy-induced nausea and vomiting, is remarkable but is based on 7 patients only [128]. CBD given orally with THC did not increase appetite or raise the quality of life in patients with cancer-related anorexia-cachexia syndrome [129]. Regarding inflammatory-related disorders, a single dose of CBD reduced aspirin-induced increased gut permeability [130], suggesting its effectiveness in disorders such as inflammatory bowel disease. On the other hand, CBD given chronically, alone or with THC, did not lead to any clinical improvement in patients with moderately active Crohn’s disease [131] or ulcerative colitis [132] (Table 2).

Although the effect of CBD on organ function has been considered only in a few clinical trials (as opposed to preclinical studies), its suitability for the treatment of some psychiatric disorders including post-traumatic stress, generalized anxiety, panic disorder and social anxiety, which may also occur in the context of COVID-19, is suggested by numerous clinical studies (for review, see [108]) (Figure 2, Table 2). CBD (given acutely or chronically) has been shown to reduce subjective anxiety and/or other reactions induced by stress (e.g., a simulated public speaking test) in healthy volunteers [133,134,135], people with naïve social anxiety disorder [136,137], patients with post-traumatic stress disorders [138], some psychiatric patients [139], persons at high risk for psychosis [140,141] and present cannabis [142,143] and past heroin users [144]. Only in a study on volunteers preselected for high paranoid traits [145] did CBD fail to attenuate anxiety. The possibility that CBD may be an effective treatment for schizophrenia has also been considered. CBD was shown to reduce positive psychotic symptoms of schizophrenia in studies by Leweke et al. [146] and McGuire et al. [147] but not in the trial by Boggs et al. [148] (Table 2).

COVID-19 is associated with painful symptoms, including myalgia, headache and abdominal or chest pain (Figure 2). Antinociceptive activity of CBD has been found in patients suffering from chronic pain [149], including pain in kidney transplant recipients [150] and in individuals with fibromyalgia [151], multiple sclerosis [152], diabetes and allodynia [153,154] but not in patients with advanced cancer [155]. In the latter studies, CBD was mainly administered in combination with THC as an oromucosal or sublingual spray (Table 2).

SARS-CoV-2 patients may develop encephalopathic symptoms ranging from alteration in consciousness to delirium, seizures and muscular damage (Figure 2). On the other hand, individuals with epilepsy and multiple sclerosis develop changes that not only increase their risk of morbidity from COVID-19 but may also mask the presentation of acute respiratory symptoms which can potentially delay the diagnosis of COVID-19. Published trials (Table 2) refer to the treatment of Dravet syndrome in children [156,157] and Lennox–Gastaut syndrome in children and adults [158,159]. Cannabidiol was administered at a dose of 10 or 20 mg/kg/day for 14 weeks and was found to reduce seizure frequency. Sativex^®^ was used as an oromucosal spray in patients with multiple sclerosis and was found to reduce spasticity [160,161,162,163]. As described above, Epidiolex^®^ (which contains a 100 mg/mL solution of CBD for oral administration) and Sativex^®^ have been approved for the treatment of intractable childhood-onset epilepsy (Dravet and Lennox–Gastaut syndromes) and as a therapy for spasticity in multiple sclerosis, respectively.

In one study [164], chronic use of CBD-enriched ointment improved skin parameters in inflammatory skin diseases (Table 2). However, Epidiolex^®^ induced a delayed skin rash in one patient with medically refractory epilepsy [165].

Importantly, as shown in Table 2, CBD, given alone or together with THC, is generally well tolerated, usually with no severe adverse events or clinical worsening. The most common side effects are nausea and vomiting, loss of appetite, diarrhea, fever and an increased concentration of aminotransferases. Even orally administered spray can produce mild to moderate unwanted effects including dizziness, nausea, diarrhea, oral pain and oromucosal disorder. Unfortunately, these side effects, to some extent, resemble the symptoms of COVID-19.

In summary, considering the effects of COVID-19 on multiple organs (Figure 2), a CBD-based pharmacotherapy that has been approved by the health authorities is limited to the treatment of some rare types of seizures and the spasticity associated with multiple sclerosis. In addition to these neuroprotective properties, anxiolytic and antinociceptive effects of CBD have been shown in clinical trials. Thus, CBD, given alone or together with THC, may be important as an adjuvant treatment to improve the well-being and quality of life of patients with COVID-19 and may even be used after recovery to limit post-traumatic stress symptoms. Further clinical studies are necessary to clarify beneficial observations obtained in small groups of patients or in cases where conflicting results have been found. Unfortunately, clinical studies (Table 2) have often failed to confirm the promising observations found in preclinical experiments (Table 1).

## 7. Opportunities, Challenges and Pitfalls of Cannabidiol Use as a COVID-19 Therapy

Cannabidiol is an interesting medicine with various pharmacological properties. Our main question is whether it is justified to recommend CBD as a therapy for COVID-19. Unfortunately, despite the identification in preclinical studies of some beneficial properties that are important for COVID-19 treatment, there are still numerous questions that need to be addressed. Preclinical and clinical effects (including their sites of action), the quality of CBD preparations, the route of administration, dosing, side effects and drug interactions are discussed in detail below.

First, the potential effects of CBD against COVID-19 may comprise the following four mechanisms.

- Numerous preclinical findings (Table 1) and reviews regarding the potential use of CBD in COVID-19 treatment [14,15,56,57,58,59,60] suggest that CBD has beneficial anti-inflammatory and antioxidative effects, which can be expected to improve the systemic symptoms that are characteristic of SARS-CoV-2 infection. Unfortunately, a comparison of the results of preclinical (Table 1) and clinical (Table 2) studies demonstrates that the favorable preclinical properties may not translate into the clinical setting (or that the appropriate clinical studies have not been conducted; see also reviews [58,61,62]). Above all, there is no confirmation of such beneficial effects of CBD with regard to its effects on respiratory failure. In addition, careful examination of whether the influence of CBD on the immune system could exacerbate viral infection is required (reviews [64,65]). Indeed, viral, fungal infections and pneumonia infections are listed among the side effects of CBD [166,167,168,169]. Moreover, it has to be considered that CBD, which was found to have a pro-oxidative effect in one preclinical study ([83]; Table 1), might even aggravate the feared cytokine storm.
- Anxiolytic and antinociceptive properties of CBD, given alone or together with THC, which have been identified in clinical studies (Table 2), suggest that it may be used as an adjuvant treatment to improve the quality of life of patients with COVID-19 and, even after recovery, may limit post-traumatic stress symptoms. However, well-designed double-blind, placebo-controlled clinical trials regarding the efficacy of CBD against COVID-19-associated panic, anxiety, depression and neurological complications are so far missing [63].
- The decreases in ACE2 and TMPRSS2 protein expression in a human tissue model are extremely interesting but so far this effect has only been shown in an in vitro study by Wang et al. [67]. Even if this mechanism was also found to occur in vivo, certain issues would have to be considered, for example, the mechanism was found for some CBD-rich extracts but did not occur when pure CBD was used. The consequence would be that an extract would need to be administered instead of a pure substance and oral administration would not be possible (for problems associated with topical administration, see below). Next, the question arises about the extent to which ACE2 and TMPRSS2 have to be decreased in order to obtain a robust antiviral effect. Moreover, it is unclear whether a reduction in ACE2 (the importance of which is highlighted in Figure 1) will lead to problems other than the struggle against the virus.
- Inhibition of the replication of SARS-CoV-2 by CBD in a cell line also represents an interesting mechanism, although this effect has only been shown in a study by Raj et al. [68] and transfer to the in vivo situation of the human body is unclear.

Second, a wide range of over-the-counter CBD-based products is now available, for example, capsules, sprays, oil droppers, gummies and plant materials to be used by smoking, dry vaporizers and e-liquid vaporizers [54]. Unfortunately, these products may be of questionable quality, are not subject to appropriate safety controls and are characterized by unknown CBD content. There is little evidence of the pharmacological activity or health benefits of non-commercial CBD preparations [54,167]. Importantly, people taking non-commercial preparations on their own should be very careful due to the possibility of dangerous consequences, such as respiratory depression following, for example, CBD gummy ingestion [170] or CBD oil overdose [171]. One should also consider that the impact of “over-the-counter” CBD on the immunity of the SARS-CoV-2 infection is unclear [62]. The use of CBD as a medication in products like Epidiolex^®^ and Sativex^®^ (see Section 3) is approved, that is, these products fulfil the essential criteria related to efficacy, safety and pharmaceutical quality.

Third, one of the most severe COVID-19 symptoms is connected with disorders of the respiratory system (i.e. ARDS). Therefore, inhalation might be a particularly appropriate route of CBD administration. The relatively high bioavailability level of ~30% (whereas oral administration leads to a value of 6% only [172]) is another argument in favor of this route of administration. However, the vaping of cannabis products (including CBD) may lead to acute pulmonary toxicity [57,173], casting some doubt on the suitability of this method of administration; it is unclear as to whether similar problems would occur if pure CBD was used instead. Since Epidiolex^®^ has been administered effectively as an oromucosal spray (Table 2), the use of a mouthwash with CBD-rich extracts might represent an interesting strategy to lower ACE2 topically [67]. It is of interest that the bioavailability of oromucosal administration of CBD is not higher than that of its oral application [172].

Fourth, with respect to dosing, most data refer to neurological disorders and this is in line with the approved indications (reviewed by Britch et al. [54]; Millar et al. [174]). Unfortunately, clinical trials dedicated to the use of CBD to treat inflammation are virtually nonexistent and clinically meaningful conclusions can therefore not be drawn [54]. A special note is necessary for Sativex^®^ (2.7 mg THC plus 2.5 mg CBD per spray). If one uses 48 sprays per day (the maximum dose used in the study of Wade et al. [160] for the treatment of multiple sclerosis), the dose of CBD will be 120 mg, corresponding to ~1.7 mg/kg. This value is much lower than that planned for use in the three CBD trials listed on ClinicalTrials.gov (2.5–7.5 mg/kg; see Section 6) or that recommended for use to treat seizures in children (10–20 mg/kg). CBD may increase the positive effects of THC and simultaneously attenuate the negative ones. A closer look, however, shows that both assumptions frequently do not hold true [175,176,177,178,179].

Fifth, CBD is generally and also according to the Critical Review Report 2018 of the World Health Organization [180], regarded as a safe compound (for review, see [54,181]). However, the authors of the latter two reviews postulated the need to conduct additional clinical trials. Indeed, various side effects of CBD have recently been described [166,167,168,169]. In addition to the increased risk of infection mentioned above, the increased tendency for respiratory depression and aspiration to occur, that is, symptoms also occurring in COVID-19, should be considered.

Sixth, CBD interacts with drug-metabolizing enzymes both of phase I (CYP3A4, CYP2C9 and CYP2C19) and phase II (uridine-5′-diphosphoglucurosonyltransferase) [181,182,183]. If CBD is used as an anticonvulsant, its combination with other anticonvulsants like clobazam or valproate might increase the risk of side effects [182,184], for example, thrombocytopenia was identified in one-third of 87 pediatric patients treated concurrently with cannabidiol and valproate [184]. Before CBD is used for the treatment of COVID-19, its possible interaction with the frontline therapy against COVID-19 should be carefully checked. The combination of CBD and glucocorticoids might lead to an increased plasma concentration of dexamethasone, since some glucocorticoids are substrates for CYP3A4 [185]. Due to the high therapeutic index of acutely administered glucocorticoids, this drug interaction may be tolerated. The situation is, however, entirely different for the antithrombin warfarin, the plasma level of which is also increased by CBD [183,186]. In this case, the interaction may lead to life-threatening bleeding.

## 8. Conclusions

**Figure 3:**
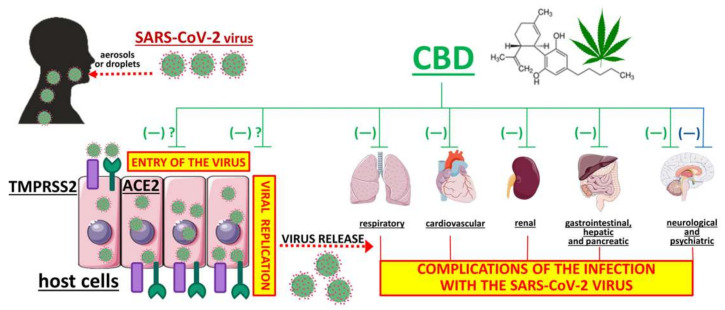
Potential therapeutic effect of cannabidiol against the SARS-CoV-2 virus infection. Three mechanisms have to be considered. The first and second mechanisms are the inhibitory effects on virus entry [67] and replication [68], respectively. These mechanisms have been described only recently and it is unclear whether they also occur in the human body. The third mechanism is a beneficial effect against complications which may also occur under a SARS-CoV-2 virus infection. In animal studies such a beneficial effect has been shown in five organ systems (green lines; Table 1) whereas in humans an effect on neurological and psychiatric disorders has been shown only (blue line; Table 2). ACE2, angiotensin-converting enzyme 2; CBD, cannabidiol; TMPRSS2, transmembrane serine protease 2.

COVID-19 is associated with multiple organ dysfunction/failure and a high mortality rate. The COVID-19 pandemic has made everyday life difficult and vaccination against SARS-CoV-2 has only just begun (and its final success is still unknown). New mutations of SARS-CoV-2 are appearing [187], so new promising therapies against COVID-19 are constantly being suggested. These include natural products, for example, CBD, a non-intoxicating phytocannabinoid from the cannabis plant with valuable pharmacological properties including strong anti-inflammatory, antioxidant, antiemetic, anticonvulsant, antipsychotic and anxiolytic properties. The broad pharmacological effectiveness and potential sites of action of CBD are shown in Figure 3. Besides its well-known antioxidant properties, downregulation of ACE2 and TMPRSS2 proteins (which are responsible for the entry of the SARS-CoV-2 virus into host cells) [67] and inhibition of SARS-CoV-2 replication [68] have only been shown very recently. Results require confirmation by independent groups and have to be demonstrated in humans in vivo. Numerous preclinical studies have shown the effectiveness of CBD in treating diseases of the respiratory system (including ARDS, one of the most dangerous symptoms of COVID-19) and its cardioprotective, nephroprotective, hepatoprotective, neuroprotective and anticonvulsant properties, that is, properties that could be beneficial for the treatment of COVID-19 (Section 5, Table 1). The beneficial influence of CBD results mainly from its significant anti-inflammatory and antioxidant properties. The anti-inflammatory properties of CBD are also responsible for a reduction in the short- and long-term consequences of viral infection, as suggested by a few in vitro and in vivo experiments (Section 4).

Unfortunately, so far, clinical studies have not confirmed the beneficial anti-inflammatory properties of CBD but it is suggested that the anxiolytic and antinociceptive properties of CBD (given alone or together with THC) may be important regarding its use as an adjuvant treatment to improve the quality of life of patients with COVID-19 and, after recovery, to limit post-traumatic stress symptoms (Section 6, Table 2). When using CBD, one should be aware of its side effects (which are rarely serious), its frequent drug interactions (which also extend to drugs used for COVID-19 treatment) and the most appropriate administration route (vaping may be effective but sometimes also dangerous). Clearly, further clinical studies are necessary to confirm the beneficial observations made for small numbers of patients, to clarify conflicting results and to broaden our understanding of the true therapeutic potential of CBD against COVID-19.

## Figures and Tables

**Table 1:** Potential use of cannabidiol (CBD) for COVID-19 treatment as suggested by preclinical studies.

	Model	Species	Dose and Route of Administration of CBD	Effects	CBD Properties Important for COVID; Mechanisms	References
**Respiratory**	ARDS induced by poly(I:C)	mouse	5 mg/kg, i.p. therapeutic, once a day for 3 days	cytokine storm and ARDS symptoms totally or partially improved (blood oxygen saturation, perivascular and peribronchiolar interstitial inflammatory infiltrate, lung fibrosis, hypertrophy and pulmonary edema) ↓IL-6 expression and ↓neutrophil frequency in the lung	anti-inflammatory	[71,72]
			5 mg/kg, i.p. therapeutic, once a day for 3 days	improvement of lung structure ↓T cells and ↑neutrophils returned towards the normal level,↑expression of apelin in the blood	anti-inflammatory regulation of apelin level	[72]
	ALI induced by LPS	mouse	1–80 mg/kg, i.p. 20 mg/kg, i.p. prophylactic; one dose before ALI induction (effects determined 1, 2 and 4 days after LPS)	lungs:↓leukocyte count; ↓leukocyte migration into lungs; ↓MPO activity; ↓vascular permeability; BALF: ↓pro-inflammatory cytokines (TNF-α, IL-6) and chemokines (MCP-1, MIP-2)	anti-inflammatory; partially dependent on adenosine A_2A_-Rs	[75]
			20 or 80 mg/kg, i.p. therapeutic; one dose 6 h after ALI induction (effects determined 24 h after LPS)	lungs: function improved: ↓resistance; ↓tissue damping and stiffness; ↓leukocyte migration into lungs; ↓MPO activity; ↓vascular permeability; BALF: ↓pro-inflammatory cytokines (TNF-α, IL-6) and chemokines (MCP-1, MIP-2)	anti-inflammatory; antagonists not used	[76]
	lung inflammation induced by LPS	mouse	75 mg/kg, p.o. prophylactic; once a day for 3 days; LPS 1 h before the last dose of CBD	lungs: ↑inflammatory changes in tissue; BALF: ↑pro-inflammatory TNF-α, IL-6, IL-23, GCSF; slight ↑inflammatory cells	pro-inflammatory; antagonists not used	[83]
	pulmonary hypertension due to monocrotaline	rat	10 mg/kg, i.p. prophylactic; once a day for 21 days	heart: ↓right ventricular systolic pressure; ↔ right hypertrophy and lung edema; ↑blood oxygen saturation; plasma: ↓leukocytes	↑blood oxygen saturation; antagonists not used	[77]
	lung injury induced by brain hypoxic–ischemic damage	newborn piglets	1 mg/kg, i.v. therapeutic; one dose 30 min after lung injury	improvement of gas exchange; ↑TLC, lungs: ↓ histological damage and edema; ↓leukocyte migration into lungs, ↓inflammatory changes; ↓vascular permeability; BALF: ↓pro-inflammatory cytokines (IL-1)	anti-inflammatory 5-HT_1A_-Rs in all parameters, except for improvement of gas exchange; site of CBD action (brain and/or lungs) unclear	[78]
	asthma induced by ovalbumin	rat	5 mg/kg, i.p. therapeutic; once a day for 2 days	serum: ↓ IL-4, IL-5, IL-6, IL-13 and TNF-α; ↔ IL-10	anti-inflammatory; antagonists not used	[79]
		mouse	5 or 10 mg/kg, i.p. therapeutic; once a day for 3 days	↓airway resistance; ↓alveolar collapse areas; ↓collagen in airways and alveolar septa; lung and BALF: ↓pro-inflammatory cytokines (IL-4, IL-5, IL-13)	anti-inflammatory; anti-fibrotic; airway resistance: CB_1_-Rs other effects: CB_1_/_2_-Rs	[80]
**Respiratory**	airway obstruction induced by ovalbumin	guinea- pig	1 mg/kg, i.v. prophylactic; one dose	↓airway obstruction induced by ovalbumin	bronchoprotective; reduction of the antigen-induced contractile responses	[81]
	cough induced by aerosolized citric acid	guinea- pig	prophylactic; aerosolized solution of 10 mg/mL for 20 minutes using a nebulizer	cough inhibition only in three out of eight animals	antagonists not used	[84]
	isolated trachea	guinea- pig	10 μM	↔ resting basal tension; ↔ contractions induced electrically or by methacholine	antagonists not used	[84]
	isolated human pulmonary artery	human	0.1–30 μM	almost full concentration-dependent vasorelaxation	endothelium-dependent vasodilatation mediated via K^+^ channels, IP, EP_4_, TRPV1 and PPARγ receptors	[82]
**Cardiovascular**	myocardial infarction induced by left coronary artery occlusion	rabbit	0.1 mg/kg, i.v. prophylactic; one dose before occlusion and one before reperfusion	heart: ↓infarct area; ↑left ventricular function; ↑blood supply to perfusion-defective region; ↓neutrophil infiltration;↓MPO activity plasma: ↓cardiac troponin I	anti-inflammatory; cardioprotective; potentiallyanti-ischemic; antagonists not used	[85]
		rat	5 mg/kg, i.p. prophylactic before occlusion and once a day thereafter for 7 days	heart:↓infarct size; ↔HR; ↓leukocyte infiltration; serum: ↓IL-6 CRP, TNF-α	anti-inflammatory; cardioprotective; potentially anti-ischemic; antagonists not used	[86]
			50 μg/kg, i.v. prophylactic; one dose before occlusion	heart: ↓infarct area; ↓arrhythmias; ↔HR	antiarrhythmic; cardioprotective; antagonists not used	[87]
			50 μg/kg, i.v. prophylactic; one dose before reperfusion	heart: ↓infarct area; ↔arrhythmias		
			50 μg/kg, i.v. prophylactic; one dose before occlusion	heart: ↓arrhythmias; ↔HR	antiarrhythmic; potential involvement of A_1_-Rs	[88]
	autoimmune myocarditis	mouse	10 mg/kg, i.p. therapeutic; once a day for 46 days	heart: improved systolic and diastolic dysfunction and myocardial stiffness; ↓left ventricular inflammatory changes; ↓necrosis; oxidative stress; ↓ fibrosis	anti-inflammatory; anti-oxidative; anti-fibrotic; cardioprotective antagonists not used	[89]
	diabetic cardiomyopathy induced by streptozotocin	mouse	1, 10 or 20 mg/kg, i.p. therapeutic; once a day for 4 or 11 weeks	heart: ↑diastolic and systolic left ventricular function; ↓oxidative and nitrative stress; ↓inflammation and NF-κB activation; ↓ fibrosis; ↓expression of pro-fibrotic genes	anti-inflammatory;antioxidative; anti-nitrative; anti-fibrotic; cardioprotective; antagonists not used	[90]
	changes in vascular endothelium function in Zucker diabetic fatty	rat	10 mg/kg, i.p. therapeutic for 7 days	mesenteric arteries: ↑endothelium-dependent vasorelaxation due to COX- or NO-mediated mechanisms; serum: ↓cardiovascular biomarkers (C-peptide, insulin and intracellular adhesion molecule-1); ↔glucose, body weight	vasoprotective; improvement in the profile of cardiovascular and metabolic parameters	[93]
**Cardiovasc.**	hypertension: primary (SHR); secondary (DOCA-salt)	rat	10 mg/kg, i.p. therapeutic; once a day for 2 weeks	↔blood pressure, HR; heart: ↓oxidative stress; ↓carbachol-induced coronary constriction; ↓left ventricular cardiomyocyte width; ↔left ventricular hypertrophy	anti-oxidative; antagonists not used	[91,92]
**Hematological**	myocardial infarction induced by left coronary artery occlusion	rat	- 50 μg/kg, i.v. before occlusion - 50 μg/kg, i.v. before reperfusion	↓platelet aggregation ↔platelet aggregation	antagonists not used	[87]
	pulmonary hypertension due to monocrotaline	rat	10 mg/kg, i.p. prophylactic; once a day for 21 days	plasma: ↓ t-PA and PAI-1	antagonists not used	[77]
	pharmacologically induced thrombus in ear venules	mouse	5 mg/kg, i.p. prophylactic; acute: one dose 30 min before thrombus induction; chronic for 3 days	acute: ↔vessel thrombogenesis chronic: ↔vessel thrombogenesis	antagonists not used	[94]
	platelet aggregation	human	in vitro, 0.1—10 μM	↔resting platelets; ↔activation induced by thrombin receptor activating peptide		
**Renal**	acute kidney injury induced by renal ischemia/reperfusion	rat	5 mg/kg, i.v. two doses before occlusion and after reperfusion	kidney:↓tubular necrosis and dilatation; ↓inflammatory changes; ↓NF-κB, COX-2, TNF-α and iNOS; serum: ↓oxidative and nitrative stress	anti-inflammatory; anti-oxidative;anti-nitrative;nephroprotective; antagonists not used	[95]
			5 mg/kg, i.a. one dose after occlusion	kidney:↓MPO activity; ↓IL-1, TNF-α and NO levels; ↓lipid and protein oxidative damage; ↔nitrite/nitrate levels	anti-inflammatory; anti-oxidative; nephroprotective; CB_1_-Rs and CB_2_-Rs expression unaltered	[96]
		mouse	10 mg/kg i.p. one dose before reperfusion	kidney: pattern of innate lymphoid cells restored to control values	nephroprotective; antagonists not used	[97]
**Gastrointestinal**	nausea or vomiting induced by lithium chloride	rat shrew	prophylactic; acute: 5 and 20 mg/kg, s.c. chronic: 5 mg/kg s.c. for 7 days	acute and chronic: ↓ nausea and/or vomiting	anti-nausea, antiemetic; 5-HT_1A_-Rs	[98]
	hepatic ischemia/reperfusion injury induced by hepatic artery and portal vein occlusion	mouse	3 or 10 mg/kg, i.p. prophylactic; one dose before reocclusion or 90 min after	serum: ↓AST and ALT; liver: ↓inflammatory changes; ↓cell apoptosis (10 mg/kg only); ↓pro-inflammatory cytokines; ↓oxidative and nitrative stress (10 mg/kg only); ↓neutrophil migration to liver tissue	anti-inflammatory; antioxidant; anti-nitrative; hepatoprotective; independent of CB_2_-Rs	[100]
	liver injury and steatosis induced by chronic ethanol administration	mouse	5 or 10 mg/kg, i.p. prophylactic for 11 days during the ethanol exposure	serum: ↓AST and ALT; liver: ↓inflammatory changes; ↓pro-inflammatory chemokines; ↓neutrophil accumulation; ↓oxidative burst of neutrophils; ↓ oxidative and nitrative stress	anti-inflammatory; anti-oxidative; hepatoprotective; antagonists not used	[101]
**Gastrointestinal**	hepatic encephalopathy due to thioacetamide	mouse	5 mg/kg, i.p. therapeutic; one dose after thioacetamide injection	liver: ↔necrosis; plasma: ↓ALT and AST, ammonia and bilirubin	partly hepatoprotective; antagonists not used	[102]
	acute hepatic toxicity induced by cocaine	mouse	30, 60 and 90 mg/kg, i.p. prophylactic; acute 30 min before cocaine injection	liver: ↓acute inflammation and damage (↓histological changes) serum: ↓ALT; ↓acute behavioral seizure	anti-inflammatory; FAAH inhibitor did not modify cocaine-induced changes in liver	[103]
	acute pancreatitis induced by cerulein	mouse	0.5 mg/kg, i.p. prophylactic; 8 doses (2 before and 6 simultaneously with cerulein)	pancreas: ↓pathological changes, ↓MPO activity in pancreas tissue; plasma: ↓amylase and lipase; ↓Il-6 and TNF-α	anti-inflammatory; pancreas-protective; possibly via GPR55 (presence in pancreas)	[104]
	inflammation and intestinal hypermotility induced by croton oil	mouse	5 and 10 mg/kg, i.p. therapeutic; one dose to mice with inflammation	intestine:↓hypermotility	involvement of CB_1_-Rs (but not CB_2_-Rs) and FAAH	[105]
	colitis induced by intracolonic dinitrobenzensulfonic acid	mouse	5–30 mg/kg, i.p. and 10–60 mg/kg, p.o. therapeutic for 3 days after colitis induction	intestine:↓hypermotility; ↔colitis; ↓colon weight and MPO activity	anti-inflammatory; antagonists not used	[106]
**Neurological**	chronic unpredictable mild stress model of depression	rat	10 mg/kg, i.p. prophylactic for 28 days	higher rate of body weight gain and sucrose preference compared to controls	prohedonic; antagonists not used	[107]
	various acute seizure models	rat mouse	one different i.v. dose dependent on the model	acute antiseizure activity	antiseizure activity	[109]
	status epilepticus—spontaneous recurrent seizures (RISESRS) model	rat	200 mg/kg for 7 weeks	↓seizure burden and motor comorbidities; reversal of the epilepsy-induced cognitive deficits		
	seizure induced by pentylene-tetrazole	mouse	60 mg/kg, i.p. prophylactic; once before induction of epileptic attack	↓seizure duration; ↓EEG changes	anti-convulsant; CB_1_, CB_2_ and TRPV1 receptors	[110]
	hepatic encephalopathy induced by thioacetamide	mouse	5 mg/kg, i.p. therapeutic; one dose after thioacetamide injection	↑neurological and cognitive functions; ↑activity; ↓activated astrocytes	procognitive; neuroprotective; antagonists not used	[102]
	sepsis-related encephalitis induced by LPS	mouse	3 mg/kg, i.v. one dose simultaneously with LPS	↑integrity of blood–brain barrier; ↓leukocyte margination in brain vessels; ↔level of oxidative stress; ↓TNF-α and COX-2	anti-inflammatory; neuroprotective	[113]
**Neurological**	perinatal hypoxic-ischemic encephalopathy induced by occlusion of carotid arteries	newborn piglet	1 mg/kg, i.v. therapeutic; one dose 30 min after induction of brain injury	brain: ↓EEG changes; ↓neuronal mortality; ↓excitotoxicity; ↓IL-1; ↓ oxidative stress	anti-inflammatory; anti-oxidative; neuroprotective partially dependent on 5-HT_1A_-Rs and CB_2_-Rs ↔brain endocannabinoid levels	[111]
			1 mg/kg, i.v. therapeutic; one dose after induction of brain injury	brain: ↑activity (EEG); ↓neuronal mortality; ↓excitotoxicity; ↓oxidative stress; ↓TNF-α; effects on excitotoxicity, oxidative stress and TNF-α additive to those of hypothermia	anti-inflammatory; anti-oxidative; neuroprotective; antagonists not used	[112]
	cerebral ischemia (stroke model) induced by MCA occlusion	mouse	0.1; 1; 3 mg/kg, i.p. two doses (before and after occlusion)	brain: ↑neurological function and motor coordination; dose-dependent ↓infarct area ↓MPO activity and ↑CBF (tested only at 3 mg/kg); blood: ↔pCO_2_, pO_2_	anti-inflammatory; neuroprotective; partially dependent on 5-HT_1A_-Rs independent of CB_1,_ CB_2_ and TRPV1 receptors	[114,115]
	encephalomyelitis induced by TMEV	mouse	180 mg/kg, i.p. twice daily starting 2 days before (prophylactic) or 3 days after infection (therapeutic)	↓acute behavioral seizures from 5 days (prophylactic) and 6 days after infection onward (therapeutic)	anti-inflammatory, anti-oxidative (not confirmed experimentally)	[73]
	multiple sclerosis induced by TMEV	mouse	5 mg/kg, i.p. therapeutic, once a day for 7 days	sub chronic effects (after 8 days): ↓transmigration of leukocytes to the nervous parenchyma by downregulating the expression of VCAM-1, CCL2 and CCL5 and the proinflammatory cytokine IL-1β and by attenuating the activation of microglia	anti-inflammatory partial involvement of adenosine A_2A_-Rs (experiments with an appropriate antagonist)	[74]
			same treatment for 10 days	chronic effects (after 80 days): improvement of motor deficits ↓microglial activation and pro-inflammatory cytokine production		
**Eye**	retinal inflammation due to LPS	rat	1 mg/kg, i.p. prophylactic before LPS treatment	↓retinal TNF-α levels	anti-inflammatory; due to A_2_-Rs but not A_1_-Rs	[117]
**Endocrine**	high-fat diet- induced obesity	rat	10 mg/kg, i.p. prophylactic for 2 weeks	plasma: ↓insulin; skeletal muscle: improved insulin signal transduction and glycogen recovery	↓lipotoxicity, leading to insulin-sensitization in myocytes; ↓expression of CB_1_, CB_2,_ TRPV1 and 5-HT_1A_ receptors	[118]
	type 1 diabetes by streptozotocin submitted to chronic cerebral hypoperfusion	rat	10 mg/kg, i.p. 30 min before and for 30 days after cerebral hypoperfusion surgery	↓body weight; plasma:↓hyperglycemia; ↑insulinemia; ↓AGEs and fructosamine; ↓dyslipidemia (LDL, HDL, TGs and total cholesterol levels); ↓AST and ALT; ↑memory performance	improvement of metabolic dysfunction; hepatoprotective; neuroprotective; anti-inflammatory	[119]
**Muscular**	Duchenne muscular dystrophy caused by dystrophin deficiency	mouse	60 mg/kg, i.p., therapeutic; three times a week for 2 weeks	muscle: prevention of the functionality loss and tissue degeneration; restoration of locomotor activity; ↓inflammation (IL-6, TNF-α); muscle strength and autophagy restored	anti-inflammatory; (involvement of TRP channels—based on in vitro experiments)	[121]
**Skin**	skin irradiated with UVA/UVB	nude rat	2.5 g in 100 g petrolatum applied to the back of rats every 12 h for 4 weeks	↓UV-induced changes in inflammation; apoptosis and oxidative stress	prevention of UV-induced metabolic changes in epidermal keratinocytes	[122]
	cultured human sebocytes and human skin organ culture	human	10 μM	↓lipogenic actions of arachidonic acid and a combination of linoleic acid and testosterone; suppression of sebocyte proliferation	anti-inflammatory (adenosine A_2a_Rs); sebostatic; lipostatic; antiproliferative (TRP4)	[123]
	experimental model of allergic contact dermatitis in keratinocytes	human	5, 10, 20 μM	inhibition of polyinosinic-polycytidylic acid-induced release of MCP-2, IL-6, IL-8 and TNF-α; no cytotoxic effect	anti-inflammatory via CB_2_ and TRPV1 receptors	[124]
**Other**	sepsis induced by cecal ligation and puncture	rat	10 mg/kg, i.p. Acute—therapeutic; one dose (after sepsis induction)	↓lipid peroxidation in lung, heart and kidney; ↓oxidative protein damage in spleen, liver and heart; ↓oxidative damage of proteins in striatum, cortex and hippocampus; ↑oxidative damage of proteins in lung	anti-oxidative; organo-protective; *pro*-oxidative in lungs	[125]
			10 mg/kg, i.p. Chronic—therapeutic; once daily for 9 days; first dose after sepsis induction	↓mortality; improvement of memory-related processes; ↓lipid peroxidation in kidney; ↓oxidative protein damage in spleen, liver, heart	anti-oxidative; organo-protective; procognitive	

The following antagonists were used to describe the multimodal mechanism of cannabidiol (CBD), namely A_2_-Rs due to blockade by ZM241385 [75,117]; A_1_-Rs by DPCPX [88]; 5-HT_1A_-Rs by WAY100635 [78,98,111,114,115]; CB_1_-Rs by AM251 [80,110] and by rimonabant [105,114,115], CB_2_-Rs by AM630 [80,110,111,114,115,124] or by SR144528 [105]; TRPV1 by capsazepine [82,114,115], SB36679 [110] or 5′-iodo-resiniferatoxin [124] and IP, EP4 and PPARγ (antagonism via L161982, Cay10441 and GW9662, respectively [82]. Moreover, some experiments were performed on knockout mice, that is, CB_2_^-/-^ [100]. 5-HT_1A_-Rs, serotonin receptor type 1A; A_1A_-R, A_2A_-R, adenosine receptor type A_1A_ and A_2A_; AGEs, advanced glycation end-products; ALI, acute lung injury; ALT, alanine transaminase; ARDS, acute respiratory distress syndrome; AST, aspartate transaminase; BALF, bronchoalveolar lavage fluid; CB-R, cannabinoid receptor; CB_1_-R, CB-R type 1; CB_2_-R, CB-R type 2; CBD, cannabidiol; CBF, cerebral blood flow; CCL2, CCL5, C-C motif chemokine ligand 2 and 5; COX-2, cyclooxygenase 2; CRP, C-reactive protein; DOCA, deoxycorticosterone acetate; DPCPX, 8-cyclopentyl-1,3-dipropylxanthine; EEG, electroencephalography; EP_4_, prostanoid EP_4_ receptor; FAAH, fatty acid amide hydrolase; GCSF, granulocyte colony stimulating factor; GPR55, G protein-coupled receptor, resembling to some extent the CB-Rs; HDL, high density lipoprotein; HR, heart rate; i.a. intraarterially; IL-n, interleukin n, for example, IL-1, interleukin 1; iNOS, inducible nitric oxide synthase; IP, prostacyclin receptor; i.p. intraperitoneally; i.v. intravenously; LDL, low-density lipoprotein; LPS, lipopolysaccharide; MCA, middle cerebral artery; MCP-1, monocyte chemoattractant protein-1; MIP-2, macrophage inflammatory protein-2; MPO, myeloperoxidase; NF-κB, nuclear factor κB; NO, nitric oxide; PAI-1, plasminogen activator inhibitor-1; pCO_2_, partial pressure of carbon dioxide; p.o. per os, orally; pO_2_, partial pressure of oxygen; poly(I:C), polyriboinosinic:polyribocytidylic acid, synthetic analogue of viral double-stranded RNA; PPARγ, peroxisome proliferator-activated receptor type gamma; SHR, spontaneously hypertensive rats; STZ, streptozotocin; TGs, triglycerides; TLC, total lung capacity; TMEV, Theiler’s murine encephalomyelitis virus-induced demyelinating disease; TNF-α, tumor necrosis factor α; t-PA, tissue plasminogen activator; TRP, transient receptor potential; TRPV*n*, transient receptor potential vanilloid subfamily member *n*; UVA and UVB, ultraviolet A and B; VCAM-1, vascular cell adhesion molecule-1. **↑**, increase; **↓**, decrease; **↔**, no change.

**Table 2:** Efficacy and safety of cannabidiol in clinical studies.

	Disease	*n*	* Study Design; Dose of CBD and THC	Application	Final Results and/or Conclusions, Properties Important for COVID-19	Profile Safety/Side Effects	References
**Respiratory**	COPD	16	* Cannabis 35 mg (THC, 18.2%, CBD, 0.1%); acute	vaporized	no effect on airway function, exertional breathlessness at rest and exercise		[126]
		9	* THC: CBD 2.7:2.5 mg/spray; maximum single dose of 4 sprays	oromucosal spray	no effect on simulated breathlessness in COPD subjects; ↓unpleasantness of breathlessness as judged by descriptors		[127]
**Gastrointestinal**	chemotherapy- induced nausea and vomiting (CINV)	7	* THC: CBD 2.7:2.5 mg/spray, <3 sprays within 2 h after chemotherapy plus <8 sprays each at days 2, 3 and 4	oromucosal spray	better protection against delayed CINV compared to standard antiemetic therapy alone	well tolerated	[128]
	cancer-related anorexia- cachexia syndrome	99	* THC 2.5 mg and CBD 1 mg; twice daily 1 h before meals for 6 weeks	p.o.	no effect on patients’ appetite or quality of life	well tolerated	[129]
	aspirin-induced increased gut permeability	10	* CBD:600 mg	p.o.	↓increased gut permeability		[130]
	moderately active Crohn’s disease	20	* CBD 10 mg/kg twice daily for 8 weeks	p.o.	no clinical improvement	excellent tolerability and safety profile	[131]
**Gastrointestinal**	ulcerative colitis	60	* 250 mg CBD-rich extract (up to 4.7% THC) twice daily before meals for 10 weeks	p.o.	no effect on ulcerative colitis but ↑quality of life outcomes	mild/moderate, mainly dizziness and somnolence	[132]
**Psychiatric**	anxiety in healthy volunteers	40	* CBD:300 mg acute	p.o.	↓anxiety to simulated public speaking		[133]
		10	* CBD:400 mg acute	p.o.	↓subjective anxiety to a simulated public speaking test, ↑mental sedation		[134]
		60	* CBD:100, 300 and 900 mg acute	p.o.	↓subjective anxiety with a dose-dependent bell-shaped curve (effective dose:300 mg only)	CBD 300 mg has a lower sedation level than clonazepam	[135]
	naïve social anxiety disorder	24	* CBD:600 mg acute	p.o.	↓subjective anxiety, ↓cognitive impairment and discomfort in speech performance; no changes in blood pressure, heart rate and skin flow	absence of psychoactive or cognitive effects	[136]
		10	* CBD:400 mg acute	p.o.	↓subjective anxiety, changes in regional cerebral flow		[137]
	stress-related disorders	11	** CBD: flexible doses, starting from 25 to 49 mg/d for 8 weeks	p.o.	↓stress-related disorders (including ↓nightmares)	well tolerated, no patients discontinued treatment due to side effects; mild side effects: fatigue, reduced concentration; gastrointestinal bloating or pain	[138]
	psychiatric patients with anxiety or poor sleep	103	CBD:25 mg/d to 50–75 mg/d; for 1–3 months	p.o.	↓anxiety in a sustained manner, ↓sleep disturbances	well tolerated, fatigue (may be related to dosing), mild sedation, dry eyes	[139]
**Psychiatric**	patients at high risk for psychosis	32	* CBD:600 mg/day for 1 week	p.o.	cortisol reaction: tended to be better;anxiety: tended to be better; ↓negative self-statement		[140]
		33	* CBD:600 mg acute	p.o.	putative antipsychotic effect by normalizing motivational salience and moderating motor response		[141]
	cannabis use disorder	128	* THC:CBD 2.7:2.5 mg/spray for 6 days, up to 32 sprays/d	oromucosal spray	↓anxiety, ↓depression, ↓craving	no differences in adverse effects between THC:CBD and placebo group	[142]
	regular cannabis users	20	** CBD:200 mg for 10 weeks	p.o.	↓depressive symptoms, ↓psychotic symptoms, ↑attentional switching, ↑verbal learning, ↑memory		[143]
	drug-abstinent patients with history of heroin abuse	42	* CBD 400 or 800 mg/d for 3 days	p.o.	↓anxiety, ↓craving, ↓HR, ↓salivary cortisol levels	no serious adverse events; mild side effects: diarrhea, headache, tiredness, fatigue	[144]
	volunteers pre-selected for high paranoid traits	32	* CBD:600 mg acute	p.o.	no benefits on anxiety or persecutory ideation		[145]
	schizophrenia	42	*** CBD; week 1: gradual increase to 800 mg/day; weeks 2–4:800 mg/day	p.o.	↓positive psychotic symptoms (no difference compared to amisulpride), mitigation of psychotic symptoms	marked tolerability / safety (with respect to weight, prolactin, hepatic or cardiac functions) compared with current medications	[146]
**Psychiatric**	schizophrenia	88	* CBD:1000 mg/d for 6 weeks on top of antipsychotic medication	p.o.	↓positive psychotic symptoms, ↓impressions and severity of illness, cognitive performance and overall functioning tended to be improved	good tolerance; diarrhea, nausea, headache, infections, insomnia; mild ↓blood pressure and moderate chest pain; no significant changes in prolactin, weight, liver function, inflammatory markers or HDL cholesterol levels	[147]
		36	* CBD:600 mg/day for 6 weeks	p.o.	no improvement of cognitive impairments and psychotic symptoms in stable antipsychotic-treated outpatients	well tolerated with no worsening of mood, suicidality or movement side effects; sedation	[148]
**Pain**	chronic pain	94	CBD-rich hemp extract ^1^ for 8 weeks	soft gels	↓chronic pain and ↑life quality (↓opioid use, ↑sleep quality)		[149]
	chronic pain in kidney transplant patients	7	CBD increasing from 50 to 150 mg twice a day for 3 weeks	p.o.	2 patients, total improvement of pain 4 patients, partial pain improvement	no serious adverse effects; dizziness, nausea, dry mouth, drowsiness, intermittent episodes of heat	[150]
	chronic pain in patients with fibromyalgia	20	* THC:CBD (mg) 1. 22.4: < 1 2. 13.4:17.8 3. < 1:18.4	single vapor inhalation	small analgesic responses	limited, such as dizziness and nausea	[151]
	neuropathic pain in patients with MS	20	THC:CBD 2.7:2.5 mg/spray for 4 weeks, 8 sprays/d	sublin. spray	↓pain rating, ↑life quality	few side effects: dizziness, nausea, dry mouth and weakness	[152]
**Pain**	peripheral neuropathic pain associated with diabetes or allodynia	380	THC:CBD 2.7:2.5 mg/spray for 38 weeks, <8 sprays per 3 h and <24 sprays every 24 h	oromucosal spray	↓pain in the majority of patients	safe and well tolerated; patients did not seek to increase their dose with time but 23% of patients ceased medication due to adverse effects (2% infections)	[153]
	painful diabetic neuropathy	30	* THC:CBD 2.7:2.5 mg/spray; dose was titrated over 2 weeks, followed by a 10-week maintenance phase (4 sprays/d)	sublin. spray	no significant improvements in pain rating and life quality		[154]
	advanced cancer patients with chronic pain	199	* THC:CBD 2.7:2.5 mg/spray: initially 4 up to 7 sprays/d	oromucosal spray	average pain score not superior to placebo		[155]
**Neurological**	Dravet syndrome in children	198	* CBD: 10 and 20 mg/kg/d for 14 weeks	p.o.	↓frequency of seizures, improvement of patients’ condition; similar effectiveness of both doses	↓appetite, diarrhea, somnolence, pyrexia, and fatigue, ↑aminotransferases in patients also taking valproate sodium; at 10 mg/kg/d better tolerance and safety profile	[156]
		108	* CBD:20 mg/kg/d for 14 weeks	p.o.	↓frequency of drug-resistant seizures	somnolence, ↓appetite, diarrhea, ↑aminotransferases in patients also taking valproate	[157]
**Neurological**	Lennox–Gastaut syndrome in children and adults	225	* CBD:10 or 20 mg/kg/d for 28 days	p.o.	↓epileptic seizures in some patients, ↓total seizure frequency, improvement of patients´ condition	somnolence, ↓appetite, diarrhea (at 20 mg/kg/d), threefold ↑aminotransferases	[158]
		171	* CBD:20 mg/kg/d for 14 weeks	p.o.	↓frequency of drug-resistant seizures	in general good tolerance of CBD as add-on therapy; mild or moderate side effects: ↓appetite, vomiting, diarrhea, somnolence, fever	[159]
	multiple sclerosis	160	* THC:CBD 2.7:2.5 mg/spray up to a maximum of 120 mg THC and 120 mg CBD/day with no more than 20 mg of each in any 3-h period for 6 weeks	oromucosal spray	↓spasticity associated with MS	good tolerance, no effects on cognition or mood; dizziness, disturbance in attention, headache, fatigue, somnolence, disorientation, feeling drunk, vertigo, application site discomfort, nausea, diarrhea, mouth ulceration	[160]
		137	THC:CBD 2.7:2.5 mg/spray up to 48 sprays/day for 21—814 days (extension study of Wade et al. [160])	oromucosal spray	↓spasticity associated with MS, clinical effect maintained after a long treatment period	serious adverse effects: seizures, fall, aspiration pneumonia, gastroenteritis; mild adverse effects: sore mouth, oromucosal disorder, oral pain, altered attention, dizziness, diarrhea, nausea	[161]
		12	THC:CBD 5.4:5.0 mg (two sublin. sprays at a 15-min interval)	oromucosal spray	↓spasticity postural and motor tests unchanged	no differences in adverse events between THC:CBD and placebo groups	[162]
		460	* THC:CBD 2.7:2.5 mg/spray; maximally 12 sprays/day for 12 weeks	oromucosal spray	↓spasticity (resistant to previous antispasticity treatment) and associated symptoms in patients with moderate to severe MS		[163]
**Skin**	psoriasis atopic dermatitis	5 5	CBD-enriched ointment, twice daily, for 3 months	ointment	in both diseases, improved skin parameters	no irritant or allergic reactions	[164]

* double-blind, placebo-controlled, randomized clinical trial; ** open-label study; *** double-blind, randomized clinical trial with active control group (amisulpride) CBD, cannabidiol; CINV, chemotherapy-induced nausea and vomiting; COPD, chronic obstructive pulmonary disease; d, day; HDL, high-density lipoprotein; HR, heart rate; MS, multiple sclerosis; *n*, number of patients; p.o. per os; sublin., sublingual; THC, Δ^9^-tetrahydrocannabinol; **↑**, increase, **↓**, decrease, **↔**, no effect. ^1^ 15.7 mg CBD, 0.5 mg THC, 0.3 mg cannabidivarin, 0.9 mg cannabidiolic acid, 0.8 mg cannabichrome and >1% botanical terpene blend.

## Data Availability

No new data were created or analyzed in this study. Data sharing is not applicable to this article.

## Supplementary Materials

The following are available online at https://www.mdpi.com/1422-0067/22/4/1986/s1 [188,189,190,191,192,193,194,195,196,197,198,199,200,201,202,203,204,205,206,207,208,209,210,211,212,213,214,215,216,217,218,219,220,221,222,223,224,225,226,227,228,229,230,231,232,233,234,235,236,237,238,239,240,241,242,243,244,245,246,247,248,249,250,251,252,253,254,255,256,257,258,259,260,261,262,263,264,265,266,267,268,269,270,271,272,273,274,275,276,277,278,279].

## Abbreviations

5-HT_1A_-R	serotonin receptor type 1A
A_1A_-R	adenosine receptor type A_1A_
A_2A_-R	adenosine receptor type A_2A_
ACE2	angiotensin-converting enzyme 2
ACE-I	angiotensin-converting-enzyme inhibitors
AGEs	advanced glycation endproducts
ALI	acute lung injury
ALT	alanine transaminase
Ang II	angiotensin II
Ang 1–7	angiotensin 1–7
AST	aspartate transaminase
ARDS	acute respiratory distress syndrome
BALF	bronchoalveolar lavage fluid
CB-R	cannabinoid receptor
CB_1_-R	cannabinoid CB_1_ receptor
CB_2_-R	cannabinoid CB_2_ receptor
CBD	cannabidiol
CBF	cerebral blood flow
CCL2, CCL5	C-C motif chemokine ligand 2 and 5
CINV	chemotherapy-induced nausea and vomiting
COPD	chronic obstructive pulmonary disease
COVID-19	coronavirus disease 2019
COX-2	cyclooxygenase 2
CRP	C-reactive protein
CVD	cardiovascular disease
d	day(s)
DOCA	deoxycorticosterone acetate
DPCPX	8-cyclopentyl-1,3-dipropylxanthine
EEG	electroencephalography
EP_4_	prostanoid EP_4_ receptor
FAAH	fatty acid amide hydrolase
GCSF	granulocyte colony stimulating factor
HDL	high density lipoprotein
HR	heart rate
i.a.	intra-arterially
IFN-γ	interferon γ
IL-*n*	interleukin *n*,e.g., IL-1, interleukin-1
IL-1β	interleukin-1ß
iNOS	inducible nitric oxide synthase
IP	prostacyclin receptor
i.p.	intraperitoneally
i.v.	intravenously
LAD	left anterior descending artery
LCx	left circumflex coronary artery
LDL	low-density lipoprotein
LPS	lipopolysaccharide
MCA	middle cerebral artery
MCP-1	monocyte chemoattractant protein-1
MERS	middle east respiratory syndrome
MIP-2	macrophage inflammatory protein-2
MPO	myeloperoxidase
MS	multiple sclerosis
NF-κB	nuclear factor κB
NO	nitric oxide
NRP1	protein neuropilin-1
PAI-1	plasminogen activator inhibitor-1
pCO_2_	partial pressure of carbon dioxide
p.o.	per os, orally
pO_2_	partial pressure of oxygen
poly(I:C)	polyriboinosinic:polyribocytidylic acid
PPARγ	peroxisome proliferator-activated receptor type γ
SARS	severe acute respiratory syndrome
SARS-CoV-2	severe acute respiratory syndrome coronavirus 2
SHR	spontaneously hypertensive rats
STZ	streptozotocin
TGs	triglycerides
THC	Δ^9^-tetrahydrocannabinol
TLC	total lung capacity
TMEV	Theiler’s murine encephalomyelitis virus-induced demyelinating disease
TMPRSS2	transmembrane serine protease 2
TNF-α	tumor necrosis factor α
t-PA	tissue plasminogen activator
TRP	transient receptor potential
TRPV1	transient receptor potential vanilloid subfamily member 1
UVA	ultraviolet A
VCAM-1	vascular cell adhesion molecule-1
