# Supplementary material for: Opportunities, Challenges and Pitfalls of Using Cannabidiol as an Adjuvant Drug in COVID-19 †
Source: Int J Mol Sci. 2021 Feb 17;22(4):1986. doi: 10.3390/ijms22041986 (PMC7922403; doi:10.3390/ijms22041986)
Supplement: Supplementary file 1 [file ijms-22-01986-s001.pdf]

Table S1. Distribution of angiotensin-converting enzyme 2 (ACE2) and possible consequences of its activation and inhibition.

	Expression Cell types	Effects of activation/ overexpression	References	Effects of inhibition/ deletion	References	ACE-2 related clinical manifestations	References
RESPIRATORY (lung)	alveolar epithelial cells, type 2 pneumocytes, tracheal and bronchial epithelial cells, macrophages, pulmonary vasculature	↓lung inflammation, ↓PAH, ↑lung endothelial function, ↓endothelial permeability, ↓lung edema, ↑pulmonary blood flow, ↑oxygenation, ↑anti-fibrotic effect, ↓cancer cell growth, ↓tumor angiogenesis and metastasis	[21, 188–196]	LUNG INJURY: ↑vascular permeability, ↑pulmonary edema, ↑neutrophil accumulation, ↑inflammation, ↑vascular remodelling, ↑endothelial dysfunction, ↑cardiopulmonary dysfunction, ↑severe lung injury	[188,191,196,-198]	ARDS/ALI, H7N9 and H5N1 influenza, lung injury, fibrosis, COPD, chronic hypoxia PAH, endotheliitis ACE2 insertion/deletion, polymorphism -↑severity of ARDS	[188,191,199–205]
UPPER RESPIRATORY ORAL/NASAL	nonkeratinized squamous epithelium of nasal, oral mucosa, and nasopharynx Goblet and ciliated cells *(not, or much less, in most olfactory receptor neurons)*	no studies addressing the impact of ACE2 on the function.		no studies addressing the impact of ACE2 on the function.		olfactory dysfunction, hyposmia, partial or total anosmia, ageusia, asthma and atopy	[206-207]

CARDIOVASCULAR\ HAEMATOLOGICAL	pericytes, endothelium of coronary arteries, cardiofibro-blasts, epicardial adipocytes, *much less in cardiomyocytes*	vasorelaxation of coronary vessels, anti-oxidative, anti-hypertrophic, anti-inflammatory, ↓cardiac remodeling, ↑postischemic heart function anti-fibrotic, antiarrhythmic, cardioprotective against: - pressure-overload–induced hypertrophy - heart failure - diabetic cardiomyopathy and nephropathy	[21,188,195, 208–213]	↑cardiomyopathy, ↑ROS, ↑myocardial hypertrophy, ↑inflammation, ↑MMP activity, age-dependent altered conductance, contractile dysfunction, ↑fibrosis, ↓coronary perfusion, ↑MI-induced cardiac dysfunction, ↑infarct size	[21, 188, 209,214–217]	acute cardiac injury, MI[1], stress-related cardiomyopathy, endotheliitis, myocarditis	[188,199,202, 216–219]
	endothelial cells of small and large blood vessels, smooth muscle cells, migratory angiogenic cells	anti-hypertensive, ↑vasorelaxation, anti-proliferative, anti-inflammatory, endothelial protection, ↓atherosclerosis	[21, 188, 195,220,221]	↑BP ↑endothelial dysfunction, ↓NO, ↑ROS, ↑VSMC hypertrophy, pro-atherogenic, pro-apoptotic, ↑permeability, microcirculation disturbances	[21,188, 195,221,222]	endotheliitis, cerebral infarction myocardial infarction ↑BP	[202,219,223–226]
	platelets	anti-thrombogenic anti-hemostatic	[226,227]	pro-thrombotic, pro-coagulant, ↓bleeding time	[226,227]	thrombosis, coagulopathy (DIC), venous and arterial thrombosis, APS	[202]

**GASTROINTESTINAL**	enterocytes (duodenum, jejunum and ileum, *but not colon*), glandular cells of gastric, duodenal, rectal epithelia, rarely in esophageal epithelium	↑nutrient (amino acid) uptake, regulation of immunity and gut microbiota ecology	[202,228]	↓tryptophan and other large amino acids in the blood, changing the microbiota profile, ↑intestinal inflammation, ↑severe colitis, pro-carcinogenic	[202,228,229]	endotheliitis, IBD, dysbiosis	[202,228–230]
**HEPATIC**	cholangiocytes *not, or much less: hepatocytes, Kupffer cells, and the endothelial lining of the sinusoids*	↓hepatic vascular resistance, ↓fibrosis in hepatocytes, ↓fibroblast proliferation, ↓sinusoid angiogenesis, anti- inflammatory, ↓hepatic gluconeogenesis, ↓hepatic insulin resistance	[16,188,232, 233]	↑inflammation, ↑ROS, ↑liver steatosis, ↓insulin sesivity, ↑fibrosis	[188,232,233]	endotheliitis, hepatitis, cholangiocyte injury, microvascular steatosis, biliary infection→injury of hepatocytes chronic liver injuries in rats and humans	[188,202,234]
**RENAL**	apical membranes of proximal tubular epithelial cells *much less: podocytes, glomerular endothelial cells, glomerular tubules*	↑natriuresis/diuresis, ↑vasorelaxation, ↑RBF, ↓glomeruli sclerosis, ↓ROS, ↓cell proliferation, anti-fibrotic, ↓albuminuria, protective in diabetes-induced kidney injury	[188,195,235]	↑fibrosis, ↑hypertrophy, ↑inflammation, ↑ROS	[21,188]	AKI, acute tubular injury, diabetic kidney disease, injured glomerular and interstitial capillaries →kidney diseases, proteinuria, haematuria	[21,195,202,226, 236–238]

NEUROLOGICAL	precursor cells of oligodendro-cytes, astrocytes (substantia nigra, cortex, brainstem and cardiovascular regulatory areas, nucleus tractus solitarii, paraventricular nucleus and rostral ventrolateral medulla), cerebral vascular endothelium	↑neurogenesis neuroprotective against ischemic and hemorrhagic strokes,<br>↑cerebral angiogenesis,<br>↓infarct volume,<br>↓neurological deficits,<br>↓loss of endothelial function of cerebral arteries,<br>↓BBB permeability,<br>↓edema,<br>↓hypertension,<br>↑cerebral blood flow,<br>↑baroreflex sensitivity,<br>↑chemoreflex, ↑Bezold-Jarisch reflex,<br>↑anti-oxidative,<br>↑anti-inflammatory,<br>↑sympatholytic,<br>↓anxiety- and depression-like behaviour,<br>↑learning and memory	[188,195,239–243]	↓neuronal survival,<br>↑inflammation,<br>↑ROS,<br>↑microglial activation,<br>↑astrogliosis,<br>↑BBB permeability,<br>↑edema,<br>endothelium dysfunction,<br>↑vascular remodelling,<br>↓baroreflex sensitivity,<br>↑BP,<br>↓cerebral blood flow,<br>↓memory and cognition,<br>↓serotonin synthesis and levels in blood and brain → ↓neurogenesis,<br>autonomic dysfunctions	[188,239,242, 243]	encephalitis,<br>edema,<br>focal degeneration of neurons,<br>stroke,<br>epilepsy	[244-247]
OPHTHALMOLOGICAL	retina, pigmented epithelial cells, rod & cone photoreceptor cells, Muller Glial cells, conjunctiva	↓diabetes mellitus–induced retinal vascular leakage,<br>↓ocular inflammation,<br>↓retinopathy,<br>↓IOP,<br>↑neuroprotection,<br>↓retinal ganglion cell death	[21,248,249]	↑diabetes-induced retinal vascular permeability,<br>↑retinal inflammation,<br>vascular remodelling, ↑ROS,<br>↓scotopic and photopic a-wave and b-wave amplitudes,<br>↑age-dependent retinal capillary loss	[250,251]	diabetic retinopathy	[252,253]

ENDOCRINE	pancreas	↑β-cell survival, ↑insulin secretion, ↓insulin resistance (diabetes), ↑β-cell proliferation, ↓β-cell apoptosis, ↑endothelial function, ↑NO, ↑vasorelaxation, anti-inflammatory, anti-oxidative, ↓microcapillary damage	[188,254]	↑insulin resistance, ↓glucose homeostasis, ↑ROS, ↑inflammation ↓β-cell proliferation	[21,254,255]	acute pancreatitis, acute diabetes, diabetic nephropathy	[219,254–256]
	adipose tissue	systemic effects on the cardiovascular system, ↑anti-inflammatory effect in EAT, ↑adiponectin, ↓lipotoxicity, ↑glucose tolerance, ↓insulin resistance, anti-obesity effect ↓fat mass but not lean mass	[21,202,211, 254,257]	phenotype of metabolic syndrome: increased abdominal adipose mass, dyslipidemia, hyperinsulinemia, leptinemia ↓insulin sensitivity, ↑glucose uptake, ↑inflammation, ↑lipotoxicity, ↑obesity-hypertension phenotype ↓weight gain in HFD males	[188,211,257, 258]	obesity-induced cardiac dysfunction	[211]

REPRODUCTIVE	ovaries,<br>oocytes,<br>uterus,<br>vagina	follicle development, oocyte maturation follicular atresia influences ovulation maintains corpus luteum progression,<br>↑estradiol, ↑progesterone (↑ steroidogenesis),<br>↑ovulation,<br>↑maturation of oocytes,<br>↑regeneration of endometrium,<br>myometrium activity,<br>↓proliferation of uterus epithelial and stroma cells,<br>↓endometrial fibrosis	[188,259–262]	↑uterus epithelial and stroma cell proliferation,<br>↑endometrial fibrosis,<br>↓ovarian follicular pool	[262,263]	infertility,<br>menstrual disorder,<br>polycystic ovary syndrome, ovarian hyperstimulation syndrome, ovarian cancer	[261–265]
	placenta, uterus and maternal-fetal interface during preg-nancy, decidual cells in arterial and venous endothelium and smooth muscle of the umbilical cord	↑anti-hypertrophic activity<br>↑uteroplacental blood flow,<br>↓preeclampsia<br>↑fetus development (myocardium growth, lungs and brain)	[266,267]	intrauterine growth restriction,<br>↑placental hypoxia, uterine artery dysfunction,<br>↓fetal growth	[268,269]	preterm birth,<br>preclampsia,<br>fetal distress	[208,270]
	testicular Sertoli and Leydig cells, spermatogonia	↑spermatogenesis,<br>↑steroidogenesis,<br>↑epididymal contractility,<br>↑sperm cell function,<br>↓ROS	[262,271]	↑inflammation,<br>↑ROS,<br>↑detachment of Sertoli cells,<br>↓Leydig cells	[262,271]	infertility,<br>↑inflammation epididymitis,<br>orchitis,<br>↓sperm quality,<br>↓hypogonadism (in obesity)	[262,271]

MUSCULAR	skeletal muscle satellite cells, mesenchymal stem cells, endothelial cells, lymphocytes	anti-fibrotic, ↓apoptosis, ↓atrophy, ↓sarcopenia, ↓insulin resistance, ↑skeletal muscle glucose uptake, ↑strength in dystrophic muscle, ↑locomotor phenotypes in muscular dystrophy	[48,188,272]	↓insulin sensivity, ↑ROS, ↑muscle atrophy, ↓muscle mass, ↓muscle strength, ↓physical performance, ↓cardiac and skeletal muscle adaptations to exercise	[48,188,231, 272]	myopathies, ↓pulmonary ventilation → ↓respiratory muscle strength, pain, weakness and injury of skeletal muscle rhabdomyolysis, sarcopenia	[48,188,273,274]
DERMATOLOGICAL	epidermal basal cell layer of skin, eccrine sweat glands, keratinocytes, *less in fibroblasts and melanocytes*	↑blood flow	[275]	↓blood flow	[275]	"skin rash", immune thrombocytopenic purpura, psoriasis, ↑inflammation, vasculitis,	[188,202,276–278]

AKI, acute kidney injury; ALI, acute lung injury; APS, antiphospholipid syndrome; ARDS, acute respiratory distress syndrome; BBB, blood brain barrier; BP, blood pressure; COPD, chronic obstructive pulmonary disease; DIC, disseminated intravascular coagulation; EAT, epicardial adipose tissue; HF, heart failure; HFD, high fat diet; IBD, inflammatory bowel disease; IOP, intraocular pressure; MI, myocardial infarction; MMP, metalloproteinases; NO, nitric oxide; PAH, pulmonary arterial hypertension; RBF, renal blood flow; ROS, reactive oxygen species; VSMC, vascular smooth muscle cells; ↑increase, ↓decrease. [1]Elevated ACE2 levels are an independent risk factor of major adverse cardiac events in patients with known coronary artery disease. Note that modulation of the renin-angiotensin-aldosterone axis (RAAS) depends on the stage of the disease. Elevated ACE2 levels have been found in chronic disease states, but ACE2 upregulation is more likely an insufficient compensatory response to overactive RAAS rather than a wrongdoer. Excessive RAAS activation and consequently the loss of ACE2 (high ACE/ACE2 ratio) are by far more important in the development and progression of diseases. [199,279].
